# Benchmark data for identifying multi-functional types of membrane proteins

**DOI:** 10.1016/j.dib.2016.05.024

**Published:** 2016-05-21

**Authors:** Shibiao Wan, Man-Wai Mak, Sun-Yuan Kung

**Affiliations:** a Department of Electronic and Information Engineering, The Hong Kong Polytechnic University, Hong Kong Special Administrative Region; b Department of Electrical Engineering, Princeton University, New Jersey, USA

## Abstract

Identifying membrane proteins and their multi-functional types is an indispensable yet challenging topic in proteomics and bioinformatics. In this article, we provide data that are used for training and testing Mem-ADSVM (Wan et al., 2016. “*Mem-ADSVM: a two-layer multi-label predictor for identifying multi-functional types of membrane proteins*” [1]), a two-layer multi-label predictor for predicting multi-functional types of membrane proteins.

**Specifications Table**

**Table:** 

Subject area	*Biology*
More specific subject area	*Bioinformatics/Computational Biology*
Type of data	*Text*
How data was acquired	*Process datasets that were obtained by searching against the UniProtKB/Swiss-Prot database with a series of stringent criteria*
Data format	*Analyzed*
Experimental factors	*Proteins were manually annotated and were extracted from UniProtKB.*
Experimental features	*For each protein sequence, its associated gene ontology (GO) information was retrieved by searching a compact GO-term database*[2], [3], [4]*with its homologous accession number.*
Data source location	*Hong Kong SAR, China*
Data accessibility	*The dataset is available with this article and**http://bioinfo.eie.polyu.edu.hk/MemADSVMServer/datasets.html*

**Value of the data**

• Knowing the functional types of membrane proteins can be helpful to elucidate the biological functions of membrane proteins.
• This article presents the first comprehensive dataset that contains non-membrane proteins, single-functional-type membrane proteins and multi-functional-type membrane proteins.
• The dataset presented here can be used as an important benchmark dataset to evaluate the performance of membrane-protein predictors.

## 1 Data

Using benchmark datasets for evaluating the performance of predictors are of great significance in various domains of bioinformatics [5], [6], [7], [8], [9], [10], such as membrane protein type prediction [11]. However, existing benchmark datasets for predicting membrane proteins are either incomplete or non-stringent. This data article describes a stringent and comprehensive benchmark dataset that comprises non-membrane proteins, single-functional-type membrane proteins and multi-functional-type membrane proteins. All of the benchmark datasets (Dataset II(C) together with Dataset I, Dataset II(A) and Dataset II(B)) are accessible from the link in *http://bioinfo.eie.polyu.edu.hk/MemADSVMServer/datasets.html*.

## 2 Experimental design, materials and methods

The dataset (we named as ‘Dataset II(C)’) here is a benchmark dataset to evaluate Mem-ADSVM [1], a webserver to identify membrane proteins and their multi-functional types. Dataset II(C) was created based on two previous datasets [5], [8], which we named as Dataset I [5] and Dataset II(A) [8]. First, we retrieved all of the 7965 non-membrane proteins in Dataset I. The procedures to create Dataset I are as follows: (1) select proteins in the UniProtKB/Swiss-Prot database; (2) exclude those protein sequences annotated with “fragment” (3) exclude those protein sequences with less than 50 amino acid residues; (4) remove those protein sequences annotated with ambiguous words, such as “by similarity”, “potential”, “probable”, etc.; (5) remove those sequences which are annotated with “membrane protein” (6) use BLASTCLUST [12] to reduce the sequence similarity to no more than 80%. The procedures for obtaining Dataset II(A) are similar to those for Dataset I except that the former collected membrane proteins instead of excluding them, and the former reduced the sequence identity to 25% instead of 80%. Because the sequence identity of Dataset I (80%) was much higher than that of Dataset II(A) (25%), we used BLASTCLUST to reduce the sequence similarity to 25%, leading to 2009 non-membrane proteins. Then, we combined these 2009 non-membrane proteins with Dataset II(A) (5307 membrane proteins) to constitute Dataset II(C) with a total of 7316 proteins, of which 7126 belong to one type, 185 to two types and 5 to three types. Specifically, the distribution of Dataset II(C) is as follows: (1) 626 single-pass type I, (2) 299 single-pass type II, (3) 42 single-pass type III, (4) 73 single-pass type IV, (5) 2437 multi-pass, (6) 403 Lipid-anchor, (7) 172 GPI-anchor, (8) 1450 peripheral and (9) 2009 non-membrane.
