# Supplementary material for: Benchmark data for identifying multi-functional types of membrane proteins
Source: Data Brief. 2016 May 21;8:105–7. doi: 10.1016/j.dib.2016.05.024 (PMC4889873; doi:10.1016/j.dib.2016.05.024)
Supplement: Supplementary file 1 — Supplementary material [file mmc1.docx]

Conflicts of interest: None.
